# Can the Policy of Increasing Retirement Age Raise Pension Revenue in China—A Case Study of Anhui Province

**DOI:** 10.3390/ijerph20021096

**Published:** 2023-01-08

**Authors:** Jin Hu, Peter-Josef Stauvermann, Surya Nepal, Yuanhua Zhou

**Affiliations:** 1 Department of Economics, Jiaxing University, Jiaxing 314000, China; 2 Department of Global Business & Economics, Changwon National University, Changwon 51140, Republic of Korea; 3 Department of Global Business Management, Gangseo University, Seoul 07661, Republic of Korea; 4 College of Business, Jiaxing Nanhu University, Jiaxing 314000, China

**Keywords:** population aging, pension revenue, delayed retirement

## Abstract

With gradual progress in the medical field and the rising living standard of people, the life expectancy of people is gradually increasing. Unfortunately, this positive development contributes significantly to the aging of societies and creates huge challenges for pension systems. In order to mitigate the pressure on its pension system in the coming years, China is considering increasing the retirement age, just like many other countries. Based on the wage data of urban employees, pension revenue and expenditure data of employees in Anhui Province over the years, we constructed a model to predict average wages and forecast the revenue of the urban pension system from 2022 to 2032. We predicted the pension revenues by simulating an adjusted retirement age under two different schemes. The results of the study showed that the policies of appropriately increasing the retirement age can raise pension revenue. Compared with a one-step retirement age change scheme, a rolling retirement age change scheme that increases the retirement age by several months each year was found to be more suitable for the healthy development of the pension system.

## 1. Introduction

When a country or region’s population aged 65 and over accounts for more than 7% of the total population, or the population aged 60 and over accounts for more than 10% of the total population, then it means that the country or region is aging as per the aging standard. According to the sampling statistics of the National Bureau of Statistics of China, as early as 2000, the elderly population in China accounted for 6.96% of the total population, and the aging of the population has become prominent. By 2021, the population aged 60 and above exceeded 267 million, accounting for 18.9% of the national population, and the population aged 65 and above accounted for 14.2% of the total population [1]. This demographic trend leads to significant challenges for China’s economy, particularly the pension and healthcare system. It must be noted that China’s main important pillar of the pension system is a pay-as-you-go (PAYG) pension system, which is implicitly a defined benefit (DB) PAYG system because it should guarantee a fixed replacement ratio. Thus, the individual pension benefit is a share of the earnings of the past. Hence, the pension benefit should remain stable as it is. Although there also exists a fully funded pillar, it is much smaller and not accessible to all employees; therefore, we ignore the latter throughout the paper. 

According to the forecast of the “Green Paper on Population and Labor: Report No. 20 on China’s Population and Labor Issues”, by the end of 2025, China’s working-age (15–64 years old) population will decline by about 30 million compared with 2019 [2]. This decline in the labor force will be associated with a sharp drop in the number of pension system contributors, and consequently, a sharp decline in China’s pension revenue.

On the other hand, with an increase in the number of elderly people in relation to the working population, pension expenditure will also increase accordingly. If relevant measures are not taken properly, the economic stability and feasibility of the existing pension system become questionable. Other things equal, the change in retirement age will affect the retirement population, revenue and expenditure of the pension system. In order to alleviate the impact of population aging on the pension system, China has initiated a plan to postpone the retirement age in 2017. The provinces Shandong and Jiangsu implemented this policy in 2022 [3]. 

In fact, many other countries apply similar policies. For example, the United States and Germany postponed the retirement age from 65 to 67, Denmark increased the retirement age to 74, and Estonia and Italy increased it to 71 (OECD 2021) [4]. The reason we focus on Anhui province is that, in China, the pension system is organized at the provincial level and not at the national level. Further, a comparison of the systems of different regions is not fruitful because of the huge economic differences between different provinces. Thus, it is quite obvious that regional comparisons between Chinese provinces do not make much sense because, at the international level, nobody would compare the pension system of Poland, which has a similar GDP to Anhui, with the pension system of Nepal, which has a similar GDP to Tibet. Thus, such comparisons would probably not deliver any useful insight in the context of our study. We should note that although every Chinese province has its own and independent pension system, the structure of the provincial pension systems is in general identical in all provinces. Therefore, an analysis and forecast of the impact of raising the retirement age on the pension revenue in Anhui Province could generally reflect and represent the situation of China as a whole.

The rest of the paper is organized as follows. In the next section, we present the related literature. In Section 3, we analyze the feasibility of delayed retirement. In Section 4, we construct an actuarial model of pension revenue in Anhui Province. We present an empirical analysis of a delayed retirement program in Section 5. In Section 6 and Section 7, we provide conclusions and policy recommendations, respectively.

## 2. Literature Review

Although the aging of societies is a common phenomenon that has been observed for decades, so far only a few papers have theoretically investigated the relationship between population aging, delayed retirement and pension funds. In addition, most of the existing literature just encompasses the normative and political-economic issues of the retirement age [5,6,7,8,9,10,11,12,13].

In the context of an aging society, it is of great significance to pay attention to the impact of an increase in retirement age on pension revenue. Shoven (2008) shows that with an increase in the proportion of the elderly population, the retirement age should be increased to avoid a shortfall in the pension system [14]. According to Jonathan and Michael (2018), a further increase in life expectancy will be expected in the presence of rapid economic development and improving health care and living environments. Consequently, the retirement age should be adjusted appropriately [15]. Wang (2016) pointed out that under the current pension policy, employees are motivated to choose to stop paying pension contributions or to retire as soon as possible [16]. Hu et al. (2022) forecasted the change in pension shortfall after the implementation of the “two-child” policy in Anhui Province in China. The prediction results revealed that the “two-child” policy cannot change the trend of increasing pension shortfall in the long run. To reduce the burden on the pension system, they recommended extending the pension contribution period or to increase the retirement age to make the pension system sustainable [17]. 

Using a three-period (childhood, adulthood, old age) overlapping generations (OLG) model (Diamond 1965), Kunze (2014) investigated the effects caused by an increasing retirement age, where population growth was exogenously given. The adults worked in the second and third periods of life, where the third period was separated into a working period and a retirement period. The time of transition from one period to the other was determined by the retirement age. Kunze (2014) found that there is an inverted U-shaped relationship between retirement age and growth [18,19]. Breyer and Hupfeld (2010) pointed out that in order to further solve the problem of the pension shortfall, the retirement age can be appropriately increased [20]. A different strand of literature used a finite horizon Blanchard–Yaari model to investigate the relationship between life-expectancy, economic growth and pensions. Kunze (2014) considered a trade-off between investing in human and physical capital and showed that an older retirement age leads to higher yields of human capital investments, which finally leads to higher growth rates and pension revenues [21,22,23]. Besides this theoretical literature, some authors tried to estimate the effects caused by an increase in the retirement age on China’s pension system and they found that an increase in the retirement age has positive effects [24,25]. Geng and Sun (2017) pointed out that when the retirement age is increased to a certain extent, it can create a secondary demographic dividend for society [26]. Deng and Yang applied the actuarial evaluation model to investigate the balance of income and expenditure of the urban employees’ basic pension fund from 2018 to 2040 and they found that the implementation of the policy of increasing the retirement age can effectively alleviate the imbalance of basic pension revenue and expenditure for urban employees [27]. 

However, some scholars put forward different views. Fanti (2014) applied a standard Cobb–Douglas production function, in the model of which the results were not unique and depended on the capital income share. If the capital share exceeds 50%, the pension benefits will decrease with an increasing retirement age [28]. Fanti (2015) used an AK production function introduced by Grossman and Yangawa (1993) and showed that an increasing retirement age leads to a decline in savings and therefore to a decline in the growth rate of incomes and pension revenues [29,30]. Lin and Lin (2016) inferred from the prediction results of the option value model that, irrespective of male workers, female workers or low-income labor groups, delayed retirement will have a certain degree of negative impact [31]. By using an overlapping generations model, Stauvermann and Hu (2018) analyzed the economic impacts resulting from an increase in life expectancy and an increased retirement age on the pension system. The results showed that the share of capital income had a crucial effect on pension revenue [32]. Romm and Wolny (2012) found that delayed retirement policy will reduce individual and aggregate savings rates and will even lead to a decline in output [33]. Martin (2009) discussed the relationship between changes in the pension system and adjustment of retirement years and found that delayed retirement could have a negative effect on society [34].

## 3. Feasibility Analysis of Increasing the Retirement Age in Anhui Province

### 3.1. Population Status of Anhui Province

According to the Statistical Yearbook of Anhui Province in 2019, the working population aged 15–64 within the province was more than 40 million, accounting for 68.18% of the total population and showing a year-on-year decrease of 3.05%. The population aged 65 and above was nearly 10 million, accounting for 12.97% of the total population, and has increased by 2.8% on a year-to-year basis. From 2010 to 2018, the median age of the population in Anhui Province increased from 36.36 years old to 40.08 years old, and the old-age dependency ratio increased from 14.21% to 19.02% [35]. The increase in the median age of the population shows the increasing trend of population aging. From the perspective of the population structure, an appropriate increase in the retirement age can effectively increase the working-age population and improve the efficiency regarding the use of human resources. 

### 3.2. The Revenue and Expenditure of Pension Systems in Anhui Province 

China’s basic pension system combines a pay-as-you-go (PAYG) part and a fully funded part. At present, Anhui Province applies a pension system as required by the central government. The revenue of the pension system is mainly composed of contributions, where the pay-as-you-go part is financed by contributions paid by employees and employers and is partly financed by government subsidies; there is a wage ceiling that is used to compute the pension contribution, where the upper limit is normally 300% of the average monthly salary of employed persons in the previous year. The fully funded part of the pension system is financed by employees’ individual contributions. Because the fully funded part has a negligible contribution toward the determination of the whole pension revenue, we did not take the fully funded part of the pension system into account. 

**Figure 1:**
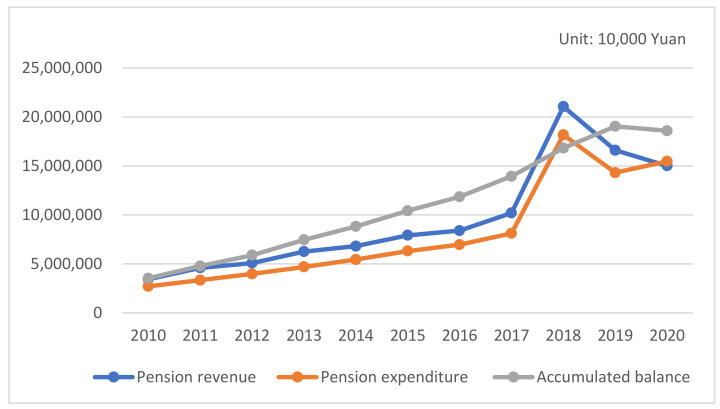
The revenue and expenditure of pension accounts in Anhui Province from 2010 to 2020.

At present, China’s legal retirement age is 60 years old for men and 55 years old for women. Due to other factors, such as illness, some people can apply for retirement as early as 45 years old, resulting in the average retirement age in society being less than 55 years old. Since 2010, the pension system’s revenue in Anhui Province has increased significantly from CNY 34.32 billion in 2010 to CNY 150.15 billion in 2020. However, at the same time, pension expenditures also increased and reached CNY 154.7 billion in 2020 [36]. Unfortunately, the growth of expenditures exceeded the growth of revenues, as indicated in Figure 1.

Developed countries have two policy options to deal with their aging populations: one is to increase the number of immigrants and the other is to increase the retirement age. In fact, European countries and Japan have already introduced policies to extend the retirement age. For example, the United Kingdom extended the retirement age to 65 for men and 60 for women. Regarding the introduction of labor from other countries, European countries implemented immigration policies that reduce skill requirements for some industries. China is still applying a retirement age system that traces back to 1951 when the average life expectancy was less than 40 years old. According to the statistics of the National Health Commission, the average life expectancy of Chinese people reached 78 years old in 2021. Confronted with the doubling of life expectancy, the appropriate adjustment of the retirement age is reasonable to some extent.

## 4. Construction of an Actuarial Model of Pension Income in Anhui Province

### 4.1. Prediction of the Insured Population

To further improve the accuracy of the prediction, this study carried out a simulation of the size of the urban population that participates in the pension system. The main variables were the size of the working-age population, the employment rate of the working population and the share of urban employees that participate in the pension system (pension coverage).

#### 4.1.1. Prediction of the Working Age Population in Anhui Province

Estimations can be executed in many different ways, for example, using the population age shift algorithm, regression analysis or the gray model. However, due to the low fitting degree of some methods and the inconsistency of some reference coefficients, we used the PADIS-INT (population macro-management and decision information system) prediction model to analyze and process the data in this study because we are convinced that is the most appropriate method given the existing data. The model has the following advantages: it is multi-functional, convenient to use and delivers results with high accuracy. However, we had to make three assumptions for the calculations: first, it was assumed that the mortality rate of the future population will not change dramatically; second, within a certain period (usually one year), the fertility rates in different age segments remain unchanged; and third, the division of regions does not change.

The prediction covered the period from 2022 to 2032 because it is probable that a pension shortfall will happen in this period. We considered a time interval of one year and applied a linear interpolation to estimate the life expectancy and the total fertility. For this purpose, we used data regarding the population of Anhui Province from statistical yearbooks. According to the laws, the urban economically active population refers to those who are 16 years old and above and who have the ability to work. Because the labor force includes the employed and the unemployed, it is necessary to accurately measure the labor employment rate. Because of the fact that male workers retire at the age of 60 years, while female workers and cadres retire at the ages of 50 and 55 years, respectively, we considered male workers aged 16–59 and female workers aged 16–54 in our study. Based on the above assumptions, we used the PADIS-INT model to predict the working-age population by gender and age in Anhui Province from 2022 to 2032 as indicated in Table 1.

**Figure 2:**
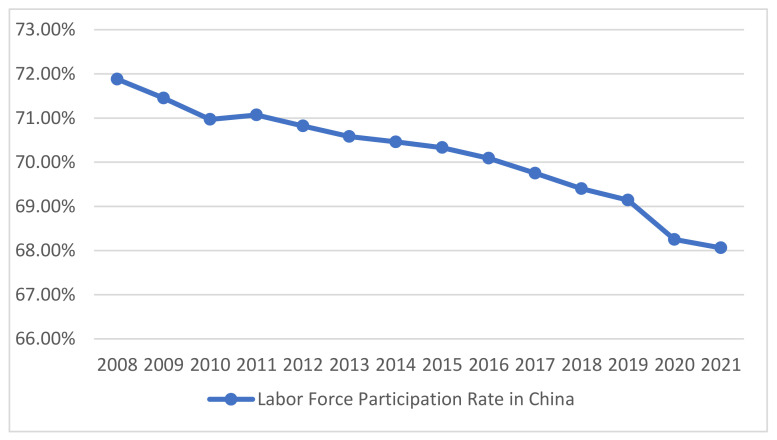
The labor force participation rate in China from 2008 to 2021.

Referring to the changes in the labor force participation rate in China from 2008 to 2021 in Figure 2, we recognized that its value dropped year by year from 71.88% to 68.06%. Therefore, according to the results of the seventh national census and assuming that the labor force participation rate will remain relatively stable in Anhui Province in the future, the labor force participation rate of the 15–64-year-old population was assumed to be 68%. With the help of this assumption, the urban economically active population of Anhui Province was predicted for the period 2022 to 2032.

#### 4.1.2. Prediction of the Participation Rate of Urban Employees

Based on the employed urban population and the pension system’s participation rate of urban employees in Anhui Province from 2008 to 2021 in Table 2, it can be stated that the overall pension system’s participation rate in Anhui Province fluctuated between 64% and 86%. After some calculations, we obtained the result that the average urban employee participation rate was 69.5% within this period. Considering that the pension participation rate rose in the past five years, it was assumed that it will increase by 4.0% annually to 93.0% by 2032, the participation rate of enterprise employees from 2022 to 2032 is shown in Table 3.

#### 4.1.3. Prediction of the Employment Rate of the Labor Force

By analyzing the employment situation of the labor force in Anhui Province from 2012 to 2021, we can state that the share of the employed population in the province increased year by year with the increase of the labor force, and the employment rate fluctuated steadily, with an overall increasing trend.

**Figure 3:**
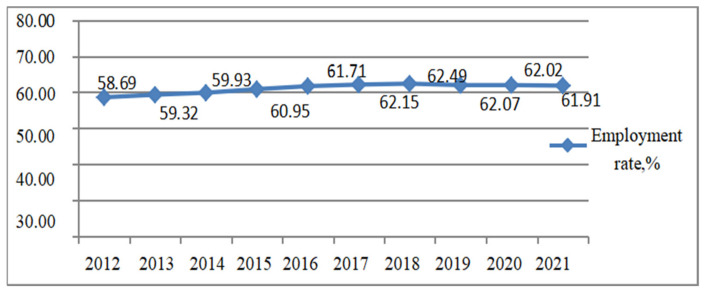
Employment rate of Anhui Province from 2012 to 2021. Data source: compiled and calculated according to the relevant data in Anhui Statistical Yearbooks from 2012 to 2021.

According to Figure 3, the employment rate in Anhui Province rose from 58.69% to 61.91% over the past ten years, with an average employment rate of 61.12%. Considering factors such as the continuous development of the economy and the introduction of relevant national policies to encourage employment, increase the years of schooling per capita, expand the employment scale and increase the demand in the labor market, it was reasonable to predict the employment rate of the labor force in Anhui Province from 2022 to 2032, as shown in Table 4.

### 4.2. Prediction of the Average Wage

#### 4.2.1. The Average Salary of Urban Workers in Anhui Province over the Years

By analyzing the average wages of employees in Anhui Province from 1999 to 2020 in Table 5, it was concluded that the average wage index fluctuated between 1.0 and 1.3, with an average of 1.13. Considering that the average wage cannot grow indefinitely, we did not use the logarithmic model method and quadratic function for fitting. Because social and economic developments experience a certain level of retardation, we used the logistic retarded growth model to forecast and analyze the average salary of workers in the province from 2022 to 2032.

#### 4.2.2. Predictions from the Retarded Growth Model

##### Building the Model

Let the average wage be *x(t)* and the growth rate of wage per capita be *r(x)*. It can be inferred that with the continuous increase of wages, the growth rate will gradually slow down, where the relationship between the two is as follows:

(1) $$\frac{dx}{dt}=r\left(x\right)x,\;x\left(0\right)=x0$$

(2) $$r\left(x\right)=r-sx\;\left(r>0,s>0\right).\;$$

Because of the influence of social and economic development, the average wage will not tend to positive infinity; therefore, let $x_{m}$ be the maximum value of the average wage such that when *x =* $x_{m}$, the growth rate *r*(*x*) = 0:

$$r-sx_{m}=0$$

Therefore, $s=\frac{r}{x_{m}}$. We inserted this result into (2) and obtained $r\left(x\right)=r\left(1-\frac{x}{x_{m}}\right)$, where $1-\frac{x}{x_{m}}$ represents the unrealized percentage of per capita wages. It can be seen that this is proportional to *r(x)*. Substituting the above results into (1), we obtained

(3) $$\frac{dx}{dt}=r\left(x\right)\left(1-\frac{x}{x_{m}}\right)$$

From this formula, we can know that when *x* increases, *r(x)* increases accordingly while $\left(1-\frac{x}{x_{m}}\right)$. decreases, and the two factors work together to increase the average wage in society. From Formula (3), we find that with the change of *x*, $\frac{dx}{dt}$ will change accordingly, in which the growth rate of *x* is at first high and then becomes lower; as t tends to positive infinity, *x* tends to $x_{m}$, with x = $\frac{x_{m}}{2}$ being the inflection point and the overall curve is an inverted “U” shape. This is because wage growth cannot be infinite, and at a certain point, the growth rate decreases and the growth rate becomes negative, which is a feature that fits well with the logistic retarded growth model. By separating the variables in the above Formula (3), we obtained the following retarded growth prediction model:

(4) $$X\left(t\right)=\frac{x_{m}}{1+\left(\frac{x_{m}}{x_{0}}-1\right)e^{-rt}}$$

##### Predicting Data

When substituting the average wage data of recent years into the above model, we used Matlab software for the calculation and obtained $x_{m}$ = 185,960 and r = 0.013; using this, we forecast the results of the average wage of employees from 2022 to 2032 in Table 6.

### 4.3. Pension Income Model

#### 4.3.1. Assumptions

We restricted our analysis to the basic pension or PAYG part of the pension system. China’s pension system is mainly composed of four parts, namely, the basic pension, enterprise supplementary pension (auxiliary pension), personal savings pension and commercial pension. The basic pension system is universal and is a strong policy implemented by the state. It has wide coverage and constitutes the most important pillar of the pension system in China. Thus, this article only considered the basic pension pillar, which is beneficial to improve the accuracy of the prediction and the feasibility of the plan. The contribution rate for the pension of urban employees is a fixed value. Beginning in May 2019, in response to the State Council’s requirement to reduce pension contribution rates, the contribution rate of the PAYG part for urban employee pension in Anhui Province was adjusted from 20% to 16%. 

#### 4.3.2. Building the Model

The revenue of the pension system can be written as follows:

(5) $$I_{t}=\beta *w_{t}*N_{t}$$

In the above formula, $I_{t}$ is the revenue of the PAYG part of the pension in year *t*, *β* is the contribution rate of the PAYG part, $w_{t}$ is the average salary of workers in year *t* and $N_{t}$ is the number of urban employees participating in the pension system in year t. As mentioned above, the contribution rate of the PAYG part in Anhui Province equals 16%, that is, *β* has a fixed value of 16%. The average wage from 2022 to 2032 was also predicted using the logistic retarded growth model above. The formula for the size of the urban insured urban population $N_{t}$ is

(6) $$N_{t}=L_{t}*\partial *\gamma$$

$L_{t}$ refers to the working-age population in year *t*, ∂ represents the employment rate of the labor force and *γ* represents the pension participation rate of urban employees. Equation (6) constitutes the total number of persons who contribute to the pension system in period *t*.

## 5. Empirical Analysis of a Delayed Retirement Program

China’s retirement age was implemented in accordance with the “Draft Rules for the Implementation of Insurance” in 1951. It has been nearly 70 years since the establishment of this draft. According to the draft, China’s retirement age can be advanced in many cases. For example, employees in special types of work and those who have lost the ability to work can apply for retirement five years in advance. In some regions, those who retire early even account for one-third of the total number of retirees. It must be noted that China has the lowest average retirement in the world. Experts from the Ministry of Human Resources and Social Security predict that by 2035, every two taxpayers will have to finance one retiree. The increase in young retirees means that as aging becomes more and more serious, China’s pension payment pressure will continue to increase. In order to further alleviate the impact of the aging population on the pension system, Hu suggestedd in 2014 [37] that it is necessary to design and formulate a policy to gradually increase the retirement age.

### 5.1. Delayed Retirement Plan I

#### 5.1.1. Scheme Design

The plan consists of two steps: the first step is to increase the retirement age of female workers and female cadres to the age of 55; the second step is to increase the retirement age of male workers to the age of 61 in 2026. This means those born in 1965 will retire at the age of 61. Then, the retirement age will be increased by two more years in 2027 and further increased until 2032 so that all men retire at age 64. Female employees will delay retirement for 8 months every year starting from 2022, where the policy shall continue for three years until 2032 so that females will retire at the age of 60. According to Table 7 and Table 8, the plan shifts the retirement age from 60 to 64 for men and from 55 to 60 for women between 2022 and 2032.

#### 5.1.2. Empirical Analysis

Using the data of young people in the labor market, along with the employment rate and pension participation rate from 2022 to 2032 predicted in the above section, according to the design of delayed retirement plan I, a forecasted estimate of the newly insured population under this plan was made. Based on the average wage predicted using the logistic retardation growth model above, we predicted the changes in the pension revenue under this retirement scheme and compared it with the revenue before the delayed retirement plan was introduced in Table 9.

From the forecasted data, we concluded that the number of contributors in the province will increase and the employees’ years they contribute will increase when the retirement age of males is delayed from 60 to 64 years old, and that of females is delayed from 55 to 60 years old. In 2022, the first year of the implementation of the plan, the pension revenue in Anhui Province will increase by CNY 188.12 million year-on-year, and up to 2032, it will be CNY 17,299.42 million higher than otherwise. Through the implementation of delayed retirement, pension income in Anhui Province is expected to increase significantly.

### 5.2. Delayed Retirement Plan II

#### 5.2.1. Scheme Design

Compared with the first plan, the second plan has a faster pace and a stronger increase in retirement age. The plan also consists of two steps. The first step is to have female workers and female cadres retire at the same age of 55; the second step is to delay the retirement of male workers for one year starting from 2023, and from then on, the retirement age will be extended for one year each time, until the retirement age of male employees will be 65 in 2032. Female employees will retire with a two-year delay from 2021, that is, those born in 1966 will retire at the age of 57 in 2023, and the retirement age will be increased in the future until retirement is delayed for eight years, that is, female employees will retire at the age of 63 in 2032. According to Table 10 and Table 11, the design delays the retirement age from 60 to 65 for men and from 55 to 63 for women between 2022 and 2032.

#### 5.2.2. Empirical Analysis

According to the retirement period design of the delayed retirement plan II, an estimate of the newly insured population from 2022 to 2032 was made. At the same time, we predicted the changes in Anhui Province’s pension revenue under plan II and we compared it with the pension revenue estimated under plan I in Table 12. 

From the table above, we concluded that plan II increased the retirement age of men from 60 to 65 and women from 55 to 63 through fast-paced reform. When comparing plan I with plan II, it was clear that the increase in pension revenue of plan II will be less than the pension revenue of plan I. It is not until 2029 that plan II starts to catch up and the pension revenue of plan II begins to exceed that of plan I. 

## 6. Sensitivity Analysis

We conducted a sensitivity analysis of the pension revenue and discuss the impact of changes in important variables on the results. Interest rate variables tended to be stable in recent years, and Liu (2013) showed that high interest rates will delay the opportunity cost and consumption utility faced by retirees. The increase in marginal loss is not conducive to delaying retirement [38]. Therefore, in this study, we only explored the influence of wage growth rate on pension revenue.

**Figure 4:**
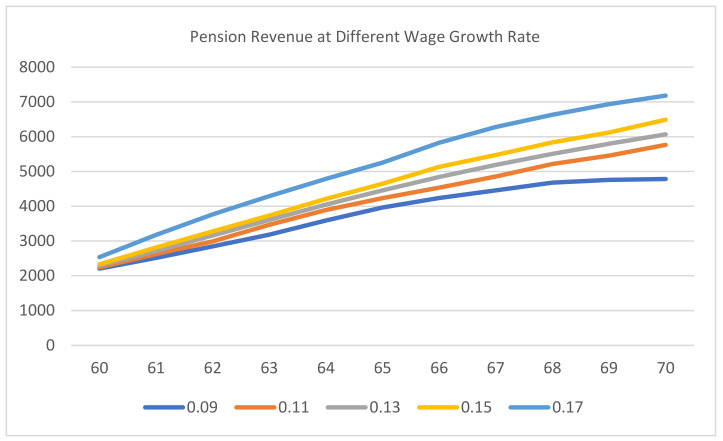
Pension revenue at different wage growth rates under plan I.

**Figure 5:**
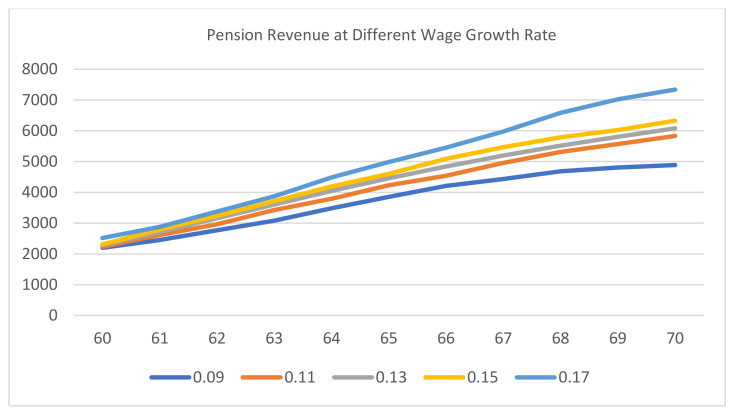
Pension revenue at different wage growth rates under plan II.

It can be observed from Figure 4 and Figure 5 that maintaining the baseline situation or a higher level of wage growth rate will increase the pension revenue of employees under both delayed retirement pension schemes. Additionally, the increase in pension revenue under plan I was higher compared with the increase in pension revenue under plan II; it was only in the later period that the pension revenue of plan II gradually caught up.

## 7. Conclusions

In this study, we used the PADIS-INT model to predict the future labor force in Anhui Province and the logistic retarded growth model to predict the average wage. In addition, an actuarial model of the pension revenue was constructed to provide a forecast for the pension revenue in Anhui Province between 2022 and 2032. We constructed two plans for increasing the retirement age and found that both retirement age change schemes will increase the pension revenue. Compared with the one-step retirement age change scheme, the rolling retirement age change policy was more suitable for the benign development of the economy. This institutional arrangement not only increased the pension revenue and delayed the early arrival of the pension fund shortfall but also enhanced the confidence level of pension participants. However, we should also notice that in many countries, the increase in retirement age for old workers induces an increase in disability pension or unemployment. These secondary effects of the delayed retirement program were not discussed in detail in the current study. In general, we believe that the increase in retirement age has more economic benefits than adverse impacts in the Chinese case. In our future studies, we will include more variables and investigate these issues. In addition, the logistic retarded model that we applied to predict the average wage is appropriate but should be tested in practice.

## 8. Policy Recommendations

We conclude that a policy that increases the retirement age smoothly can tackle the problem of the pension system by increasing pension revenues. Based on our study, we propose implementing a step-by-step increase of the retirement age, with the female’s retirement age postponed first and then the male’s. In China, the current retirement policy has been implemented for almost 70 years. The fact that, for most people, accepting the extension of the retirement age requires an adaptation process must not be overlooked. On the one hand, policy arrangements should be formulated in advance for the gradual extension of the retirement age. On the other hand, some additional rewards could be given to employees who voluntarily apply for postponing retirement to stimulate the active participation of the whole society.

Employment protection measures are also of high importance and should be taken into utmost consideration by policymakers. The actions of the aging population can have a certain impact on the employment of the young population, their actions either constitute continuing their current jobs, changing their jobs or retiring from their jobs. Compared with the young, it is difficult for elderly workers to endure high-intensity work. Thus, it may lead to a reduction in work efficiency and increase the cost of employment for the company. Therefore, to maintain a balance in the employment opportunities of young workers and old workers, the government needs to formulate corresponding macro-incentive policies to protect the common interests of workers, enterprises and society. On one hand, suitable positions for the employment of the elderly population should be set up and certain tax relief or financial subsidies should be provided to recruiting enterprises. On the other hand, some insurance premiums should be reduced or exempted to a certain extent for older workers who are actively employed. Moreover, investment in the elderly care market should be increased, which not only increases human capital but also stimulates consumption, thereby promoting continuous economic development.

To further improve the structure of the pension system, a delayed retirement policy needs to be formulated, which can eventually raise the pension revenue. Moreover, various measures should be adopted to expand the financing channels of pension funds. The provision of a stable and safe operating environment for the pension investment market should be ensured to allow for more professional investment operations, which can reduce the volatility risk of pension funds. Furthermore, the aim should be to increase the participation rate and improve the comprehensive coverage of the pension system. A way to realize the former is to offer cost-free childcare centers for young children. 

## Figures and Tables

**Table 1:** The sizes of the male and female labor forces in Anhui Province from 2022 to 2032 (unit: person).

Year	Males Aged 16–59	Females Aged 16–54	Total Working Labor Force
2022	196,801	181,267	378,068
2023	198,122	179,522	377,644
2024	199,430	177,381	376,811
2025	200,547	174,627	375,174
2026	201,439	172,019	373,458
2027	201,943	169,470	371,413
2028	201,636	167,132	368,768
2029	200,812	164,856	365,668
2030	199,392	162,638	362,030
2031	198,050	160,957	359,007
2032	196,633	159,675	356,308

**Table 2:** Anhui Province pension participation rate over the years.

Year	Employed Population of Urban Workers (Ten Thousand People)	Insured Population	Participation Rate
2008	901.9	578.41	64.13%
2009	936.2	628.15	67.10%
2010	973.5	669.5	68.77%
2011	1038.3	729.27	70.24%
2012	1141	783.76	68.69%
2013	1226.2	811.33	66.17%
2014	1277.4	829.25	64.92%
2015	1292.1	857.51	66.37%
2016	1327.5	892.24	67.21%
2017	1378.5	1076.97	78.13%
2018	1385.3	1141.72	82.42%
2019	1415.5	1188.74	83.96%
2020	1515.9	1283.55	84.67%
2021	1605.4	1384.2	86.22%

Data source: calculated based on the 2008–2021 data in Anhui Statistical Yearbooks.

**Table 3:** Assumptions of the basic pension participation rate of enterprise employees from 2022 to 2032.

Year	2022	2023	2024	2025	2027	2027
Participation rate	86.56%	86.91%	87.26%	90.75%	91.11%	91.48%
Year	2028	2029	2030	2031	2032	
Participation rate	91.84%	92.21%	92.58%	92.95%	93.32%	

**Table 4:** Assumptions of the employment rate of the labor force in Anhui from 2022 to 2032.

Year	2022	2023	2024	2025	2026	2027
Employment rate	62.27%	62.64%	63.01%	63.39%	63.77%	64.14%
Year	2028	2029	2030	2031	2032	
Employment rate	64.53%	64.91%	6530%	65.69%	66.08%	

**Table 5:** Average wages in Anhui Province from 1999 to 2020.

Year	Average Wage	Average Wage Index	Year	Average Wage	Average Wage Index
1999	6516	1.00	2010	33,341	1.12
2000	6989	1.07	2011	39,352	1.18
2001	7908	1.13	2012	44,601	1.13
2002	9296	1.18	2013	47,806	1.07
2003	10,581	1.14	2014	50,894	1.06
2004	12,928	1.22	2015	55,139	1.08
2005	15,334	1.19	2016	59,102	1.07
2006	17,949	1.17	2017	65,150	1.10
2007	22,180	1.24	2018	74,378	1.14
2008	26,363	1.19	2019	79,037	1.09
2009	29,658	1.12	2020	85,854	1.11

Data source: based on the relevant data in Anhui Statistical Yearbooks from 1999 to 2020.

**Table 6:** Average wage forecast for Anhui Province from 2022 to 2032.

Year	2022	2023	2024	2025	2026	2027
Average salary (CNY/year)	100,102	113,667	126,891	139,156	149,991	159,103
Year	2028	2029	2030	2031	2032	
Average salary (CNY/year)	166,438	172,113	176,353	179,428	181,601	

**Table 7:** Delayed retirement plan I (male workers).

Year of Birth	Age at 2025	Delay Retirement	Actual Retirement Age	Year of Retirement
1965	60	1 year	61	2026
1966	59	2 years	62	2028
1967	58	3 years	63	2030
1968	57	4 years	64	2032

**Table 8:** Delayed retirement plan I (female workers).

Year of Birth	Age at 2022	Delay Retirement	Actual Retirement Age	Year of Retirement
1967	55	8 months	Age 55 + 8 months	2022
1968	54	16 months	Age 56 + 4 months	2024
1969	53	2 years	Age 57	2026
1970	52	32 months	Age 57 + 8 months	2027
1971	51	40 months	Age 58 + 4 months	2029
1972	50	4 years	Age 59	2031
1973	49	56 months	Age 59 + 8 months	2032

**Table 9:** Pension revenue of Anhui Province (deferred retirement plan I).

Year	Newly Insured Population	Pension Revenue before Increasing Retirement Age (100 Million CNY)	Pension Revenue after Increasing Retirement Age (100 Million CNY)	The Difference before and after Increasing Retirement Age (Ten Thousand CNY)
2022	377,076	2241.01	2242.86	18,496.77
2023	378,619	2653.91	2658.68	47,660.99
2024	382,070	3084.34	3094.04	96,985.71
2025	382,086	3515.32	3531.35	160,305.34
2026	391,938	3931.55	3959.15	275,983.56
2027	393,785	4320.77	4362.57	417,987.86
2028	401,054	4676.14	4737.25	611,078.49
2029	402,040	4995.72	5078.67	829,516.53
2030	404,674	5281.23	5389.34	1,080,935.6
2031	407,919	5537.24	5673.62	1,363,769.82
2032	414,946	5768.95	5938.25	1,692,992.76

**Table 10:** Delayed retirement plan II (male workers).

Year of Birth	Age in 2023	Delay Retirement	Actual Retirement Age	Year of Retirement
1963	60	1 year	61	2024
1964	59	2 years	62	2026
1965	58	3 years	63	2028
1966	57	4 years	64	2030
1967	56	5 years	65	2032

**Table 11:** Delayed retirement plan II (female staff).

Year of Birth	Age in 2021	Delay Retirement	Actual Retirement Age	Year of Retirement
1966	55	2 years	57	2023
1967	54	4 years	59	2026
1968	53	6 years	61	2029
1969	52	8 years	63	2032

**Table 12:** Pension revenue in Anhui Province (deferred retirement plan II).

Year	Newly Insured Population	Pension Revenue in Scheme I (100 Million CNY)	Pension Revenue in Scheme II (100 Million CNY)	Pension Revenue Difference (Ten Thousand CNY)
2022	370,002	2242.86	2240.37	−248.58
2023	377,679	2658.68	2656.31	−236.84
2024	382,542	3094.04	3092.19	−184.97
2025	384,837	3531.35	3529.61	−173.22
2026	397,182	3959.15	3658.00	−115.48
2027	395,660	4362.57	4361.78	−78.29
2028	398,309	4737.25	4737.07	−17.62
2029	409,526	5078.67	5078.99	31.32
2030	412,150	5389.34	5390.01	66.55
2031	408,998	5673.62	5674.34	72.42
2032	424,726	5938.25	5939.45	120.38

## Data Availability

Data are available from the corresponding author upon reasonable request.
